# A unique regulated cell death-related classification regarding prognosis and immune landscapes in non-small cell lung cancer

**DOI:** 10.3389/fimmu.2023.1075848

**Published:** 2023-02-03

**Authors:** Wei Su, Ting Hong, Baijie Feng, Zhou Yang, Guang Lei

**Affiliations:** ^1^ Department of Radiation Oncology, Hunan Cancer Hospital and the Affiliated Cancer Hospital of Xiangya School of Medicine, Central South University, Changsha, Hunan, China; ^2^ Department of Gynecology Oncology, Hunan Cancer Hospital and the Affiliated Cancer Hospital of Xiangya School of Medicine, Central South University, Changsha, Hunan, China; ^3^ Department of Medical Oncology, Fudan University Shanghai Cancer Center, Shanghai Pudong Hospital, Shanghai, China; ^4^ Department of Oncology, Shanghai Medical College, Fudan University, Shanghai, China; ^5^ Department of Oncology, Shanghai Pudong Hospital, Fudan University Pudong Medical Center, Shanghai, China; ^6^ Department of Head and Neck Surgery, Fudan University Shanghai Cancer Center, Shanghai, China

**Keywords:** regulated cell death, non-small cell lung cancer, tumor microenvironment, immunotherapy, immune checkpoint inhibitors

## Abstract

Regulated cell death (RCD) contributes to reshaping the tumor immune microenvironment and participating in the progression of non-small cell lung cancer (NSCLC); however, related mechanisms have not been fully disclosed. Here, we identified 5 subclusters of NSCLC based on consensus clustering of 3429 RCD-associated genes in the TCGA database and depicted the genomic features and immune landscape of these clusters. Importantly, the clusters provided insights into recognizing tumor microenvironment (TME) and tumor responses to immunotherapy and chemotherapy. Further, we established and validated an RCD-Risk model based on RCD-associated genes, which strongly predicted the prognosis, TME, and immunotherapy outcomes in NSCLC patients. Notably, tissue microarray staining confirmed that the expression of LDLRAD3, a core gene in RCD-Risk model, correlated with poor survival. In conclusion, we developed a novel RCD classification system and RCD-Risk model of NSCLC, serving as a robust and promising predictor for prognosis and immunotherapy benefit of individual NSCLC patients.

## Introduction

Non-small cell lung cancer (NSCLC) accounts for 80% to 85% of all pathology types of lung cancers, including lung squamous cell carcinoma (LUSC), lung adenocarcinoma (LUAD), and large cell carcinoma. NSCLC is a highly aggressive cancer type with an extremely low response to chemotherapy. Immune checkpoint inhibitors (ICIs), also known as immunotherapy, have emerged as breakthrough treatments for advanced NSCLC. These drugs target inhibitory interactions between immune checkpoint receptors and their ligands, such as the programmed death 1 (PD-1) pathway. While the addition of immunotherapy has changed the mode of NSCLC treatment and improved the survival of patients; however, part patients cannot obtain the same degree of clinical benefit and face potential adverse reactions. Biomarkers predictive of ICI response include target antigen expression (e.g., PD-1/PD-L1), tumor mutational burden (TMB), DNA mismatch repair deficiency/microsatellite instability, and tumors at baseline T cell infiltration. However, potential responder populations outside the recommended PD-1 expression were excluded from first-line immunotherapy due to the lack of alternative predictive biomarkers. Accurate identification of immunotherapy responders could expand the eligible population for immunotherapy, thereby reducing the burden of chemotherapy toxicity in NSCLC patients.

Regulated cell death (RCD) is closely related to tumor development and treatment outcome. Tumors suffer different death modes under different conditions. Necrosis and apoptosis are two most classical cell death processes. Necrosis refers to the passive death of cells caused by physical damage, chemical damage, mechanical damage, poisons, microorganisms, radiation, etc. Apoptosis refers to the active and orderly cell death generated by gene regulation in order to maintain the homeostasis of the body under physiological or pathological conditions. Recently, more and more cell death types were proposed. Netotic cell death (NETosis) is a Neutrophil Extracellular Traps (NET) driven form of cell death that is regulated by NADPH oxidase-mediated ROS production and histone citrullination. Pyroptosis is a form of cell death activated by the inflammasome and plays an important role in inflammation and immunity. Ferroptosis is a form of cell death driven by iron accumulation and lipid peroxidation. These cell death processes have close association with NSCLC by regulating progression, immune microenvironment, chemoresistance and so on. Several studies have revealed the interaction between RCD and antitumor immunity. Targeted therapy against autophagy, ferroptosis, pyroptosis and necroptosis combined with immunotherapy may exert powerful antitumor activity, even tumors resistant to ICIs.

In this research, based on 999 cases of NSCLC patients in the TCGA database, we developed a novel classification system through RCD-associated genes. We divided all NSCLC cases into five clusters with different genomic features. Notably, these clusters showed different response to immunotherapy and chemotherapy. Further, we established and validated an RCD-Risk scoring model for predicting prognosis and immunotherapy outcomes of NSCLC patients. Notably, our immunohistochemical staining in tissue microarray confirmed that the expression of LDLRAD3, a core gene in RCD-Risk model, was associated with poor survival. Overall, our study provides insights to understand the distinct prognosis and immune landscapes between NSCLC subclusters and presents a robust model to stratify NSCLC patients for optimal immune response and survival.

## Material and methods

### Data acquisition and assessment

999 cases of NSCLC multi-omics data were acquired from the TCGA database, including transcriptional data, somatic mutation data, copy number variation data, DNA methylation and clinical information (**Table S1**). Other 115 cases (GSE26939) and 146 cases (GSE30219) of NSCLC were derived from GEO database, respectively (**Table S2**) (1, 2). Samples with missing survival data were removed. 295 ferroptosis associated genes, 19 NETosis associated genes, 1809 aging associated genes, 1972 necroptosis associated genes, 57 pyroptosis associated genes, 18 extrusion death associated genes, 1972 necrosis associated genes and 222 autophagy associated genes associated genes were acquired from FerrDb database (http://www.zhounan.org/ferrdb), HADb database(http://www.autophagy.lu/clustering), and several published studies (**Table S3**) (3–5).

### Classification of NSCLC based on RCD-related genes, GSVA, and somatic mutation or CNV or DNA methylation analysis

Based on the expression matrix of cell death associated genes, unsupervised clustering was performed using the R package ConsensusClusterPlus (v1.50.0). Then, the R packages survival (v3.2-7) and survminer (v0.4.8) were applied for survival analysis. Gene set variation analysis (GSVA) was performed using Hallmark gene set to obtain enriched pathways in different clusters. The landscape of somatic mutation was assessed and visualized by R package maftools (v1.0-2). The landscape of DNA methylation was assessed by R package ChAMP (v2.16.2). P.Val < 0.01 & |logFC| > 0.2 were used as thresholds to screen differentially methylated sites. Chi-square tests were performed to assess copy number variation (CNV) of cell death genes using p<0.05 as a threshold.

### Characterization of immune landscape and immunotherapy/chemotherapy responses

The xCELL algorithm was used to calculate the proportion of immune cells in all samples. Infiltration matrix of immune cells in NSCLC patients downloaded from TIMER2.0 database (6, 7). For immune function scores, enrichment levels of immune function-related gene sets per sample were quantified by the ssGSEA (single-sample gene set enrichment analysis) algorithm in the R package ‘GSVA’ package.

The R package ESTIMATE (v1.0.13) was further used to calculate the StromalScore, ImmuneScore, ESTIMATEScore, and TumorPurity of all samples in the TCGA-NSCLC data.

For immunotherapy or chemotherapy responses, SubMap analysis was used to map NSCLC samples to melanoma samples with inhibitor treatment information, and the similarity between samples was calculated to predict the response of NSCLC to immune checkpoints inhibitors. The treatment response data of chemotherapy was acquired using the R package TCGAbiolinks (v2.16.4). We then further calculated the distribution differences of treatment response groups in different clusters.

### Differentially expressed genes screen and survival analysis

The R package limma (v3.42.2) was used to screen differential cell death-related genes between normal and tumor samples with P.Val <0.05 & |logFC| > 1 as the threshold. For survival analysis, abnormal samples with survival times below 0 days were removed, and genes associated with cell death pathways with variance < 0.2 were filtered out, followed by batch cox univariate regression analysis of TCGA data using the R packages survival and survminer. After regression analysis, genes significantly associated with OS were screened at a threshold of p < 0.05 for subsequent analysis.

### Establishment of risk scoring model *via* Lasso/Cox analysis

Lasso regression was performed to reduce the dimensionality of the univariate cox regression results. Repeated iterations were performed by 500 Lasso regressions, thus eliminating contingency. Those with more than 400 occurrences in 500 iterations were selected for model construction using R package glmnet (v4.0-2). Next, the model was constructed by multivariate Cox regression, and genes were included in the model by increasing stratum by stratum. The AUC of model was calculated for all gene combinations, and the model with the highest AUC value was selected as the final. Finally, 53 regulated cell death (RCD)-related genes were screened as the input to the final model, and calculated the risk score of each sample based on the following formula:


$${\text{RScore}}_{i}=\;\sum_{j=1}^{n}\exp_{ji}\times {\text{β}}_{j}$$

In the formula, exp represents the expression of the corresponding gene, β represents the regression coefficient (coef) of the corresponding gene in the lasso regression results, RScore represents the expression of the significantly related genes in each sample multiplied by the coef of the corresponding gene and then summed, i represents the sample, j represents the gene.

### Validation of model performance

Based on the median of risk score, we divided NSCLC cases into the high and low risk groups, respectively. We drew the Kaplan-Meier curve and ROC curve in combination with the OS data and calculated the p-value. p-value < 0.05 was considered as a significant difference between high and low risk groups. Sample risk scores are used as model predictions, combined with the survival data to calculate the AUC value of the model and to plot the time-based ROC curve. AUC values greater than 0.7 at 1, 3, and 5 years indicate good model efficacy.

### Prediction of immunotherapy outcomes

For calculation of tumor immune dysfunction and exclusion (TIDE) scores, we first performed two-direction median centered correction on the expression profile data of NSCLC from TCGA and uploaded the corrected expression profile matrix to the TIDE database online website (http://tide.dfci.harvard.edu/), to obtain the patient’s TIDE score.

For assessment of immunotherapy benefits, GSE135222 data were downloaded from the GEO database. Kaplan-Meier curves were plotted between high and low risk groups to demonstrate survival differences, and box plots were drawn to demonstrate differences in immunotherapy response between high and low risk groups.

### Calculation of tumor mutational burden

TMB refers to the number of somatic non-synonymous mutations in a specific genomic region, usually expressed in mutations per megabase (mut/Mb). TMB can indirectly reflect the ability and degree of tumor production of neoantigens and predict the immunotherapy efficacy of various tumors. We downloaded TMB data of NSCLC patients through the R package ‘TCGAmutations’ with the parameter set to pipelines = “mutect2”. Differences in TMB between high and low risk groups were calculated using the wilcoxon test.

For TMB combined with RCD-Risk, samples were divided into four groups based on risk value and TMB: high TMB with high risk value; high TMB with low risk value; low TMB with high risk value; and low TMB with low risk value, and survival analysis was performed on NSCLC samples using the R package survivor and survminer.

### Prediction of favorable drugs in high and low risk groups and drug sensitivity screening

Drug sensitivity was predicted by genes expression profiles in cell lines using the calcPhenotype in the R package ‘oncoPredict’. IC_50_ of drugs was predicted by ridge regression model established by gene expression profiles in cell lines of GDSC (www.cancerrxgene.org) and TCGA database using pRRophetic algorithm (8, 9).

The favorable drugs were predicted by CMap using eXtreme Sum (XSum) algorithm. The criteria for identifying the molecular signature of a disease were |Log2FC| > 0.5, p < 0.05. Based on CMap’s theory, the lower the score, the more likely this drug is to reverse the molecular signature of the disease and theoretically more likely to have the ability to treat the disease (10).

### Immunohistochemistry

Tissue microarray of NSCLC specimens (RLN121e) was obtained from Boruilin Biotechnology Co., Ltd. (Xi’an, China) and used to validate the correlation between LDLRAD3 expression and NSCLC survival. Detail features were showed in **Table 1**. Fixing specimens with 10% neutral formaldehyde at room temperature for 12 hours was immediately performed on the collected specimens. Sections of the fixed specimens were cut into 4 m thick paraffin blocks. LDLRAD3 antibody (1:200, Bioss Biotech, Inc. Beijing, China) was incubated overnight at 4°C, and anti-rabbit immunoglobulin G (ab205718;Abcam;1:200) was incubated at 37°C for 20 minutes. Finally, the sections were visualized under a light microscope or scanned by the NanoZoomer S360 Slide Scanner (Hamamatsu Photonics, Japan).

**Table 1:** Clinical features of NSCLC patients.

Characteristics	No.	Expression of LDLRAD3		P-value
		Low (n=52)	High (n=52)	
Gender				0.242
Male	70	39	31	
Female	34	13	21	
Age				0.4051
<60	62	29	33	
≥60	42	23	19	
Pathology diagnosis				0.1614
Squamous cell carcinoma	52	28	24	
Adenocarcinoma	52	24	28	
Pathological Stage				0.4178
I–II	91	45	46	
III–IV	13	7	6	
Grade				ns
1	3	1	2	
2	46	26	20	
3	55	25	30	

ns, no significance.

### Cell culture and transfection

NSCLC cell lines A549 and NCI-H1299 were obtained from the University of Colorado Cancer Center Cell Bank and cultured in RPMI1640 medium supplemented with 10% FBS (Invitrogen, Carlsbad, CA, USA) at 37°C in a 5% CO_2_ atmosphere.

LDLRAD3 overexpression vector was constructed by PCR LDLRAD3 cDNA into the pCDH-CMV. Lipo3000 transfection reagent (Invitrogen, Inc.) was used to cotransfect HEK293T cells with the target plasmid or the negative control vector, psPAX2, and PMG.2G to obtain LDLRAD3 overexpression lentivirus or negative control lentivirus. Then, the A549 and NCI-H1299 cells were infected with the lentivirus (multiplicity of infection, MOI = 10). The LDLRAD3 overexpression cell lines A549-OE, NCI-H1299-OE and the negative control cell lines A549-NC and NCI-H1299-NC were screened by puromycin (2 μg/mL, 72 h). Finally, the protein expression of LDLRAD3 was tested by Western blotting in NC and OE groups.

### Cell proliferation assay

For this assay, five thousand cells were seeded into 96-well plates and cultured for 0 h, 24 h, 48 h and 72 h. Prior to the assay, 10 µl of Cell-Counting Kit-8 (CCK-8; Dojindo Molecular Technologies, Japan) solution was mixed with 100 µl medium in each well of 96-well plate, and incubated for 2 h. Finally, the absorbance of each well at a 450 nm was measured.

### Colony formation assay

First, 500 cells were inoculated in 6-well plates and incubated at 37°C, until more than 50 cells were available for most clones. This was followed by staining with 0.2% crystalline violet solution for half an hour at room temperature and washing with PBS three times. Then these plates were captured with a 4X light microscope and (77002; Yuyan Instruments Co., Ltd. Shanghai, China).

### Cell migration and invasion assays

Transwell plates (24-well insert, 8 μm pore size; BD Biosciences, Bedford, MA, USA) were used to perform cell migration and invasion assays. The filters (Corning, USA) were coated without (migration) or with (invasion) 55 μl Matrigel (1:8 dilution; BD Biosciences). For the migration assays, 2 × 10^5^ cells were diluted to 200 μl with serum-free medium and seeded into upper chambers uncoated with Matrigel. Then, 500 μl of complete medium was added to the lower chamber as a chemoattractant. After 2 days of incubation at 37°C, the chambers were fixed with 4% paraformaldehyde for half an hour and then stained with 0.1% crystal violet for half an hour at room temperature. For invasion assays, 2 × 10^5^ cells were diluted to 200 μl with serum-free medium and seeded into Matrigel-coated upper chambers. The remainder of the trial protocol was the same as described above. Five different areas of each replicate filter were counted and photographed under an inverted microscope.

### Statical analysis

Most of the data were analyzed and visualized by R software using different packages. Except for detail analysis methods described above, the Kruskal-Wallis or Wilcoxon test was applied in analyzing differences between pathway enrichment, clinical feature distribution, immune infiltration, and therapy response.

## Results

### Genotyping and clinical features of NSCLC based on cell death-related genes

**Figure 1:**
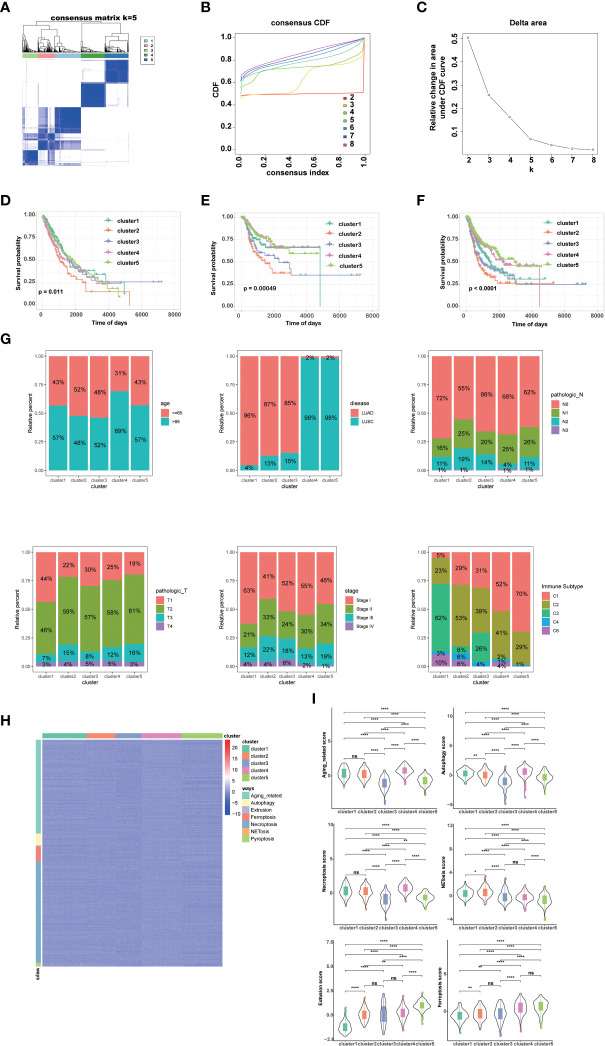
Genotyping and clinical features of NSCLC based on cell death-related genes. **(A)** Heatmap depicts consensus clustering solution (k = 5) for on cell death associated genes in 999 NSCLC patient samples. **(B, C)** Delta area curve of consensus clustering indicates the relative change in area under the cumulative distribution function (CDF) curve for k = 2 to 8. **(D)** Kaplan–Meier curves of overall survival in indicated clusters. **(E)** Disease free interval of different clusters *via* Kaplan-Meier analysis. **(F)** Progression free interval of different clusters *via* Kaplan-Meier analysis. **(G)** Differences in age, disease histologic subtypes (LUAD, lung adenocarcinoma; LUSC, lung squamous cell carcinoma), pathologic N (lymph nodes) stages, pathologic T (tumor) stages, TNM stages, and immune subtypes among 5 NSCLC clusters. **(H, I)** Differences in cell death associated pathways **(H)** and scores **(I)** among 5 NSCLC clusters. (****p<0.0001; **p<0.01; *p<0.05; *ns*, no significance.).

By performing unsupervised clustering analysis using the expression matrix of cell death-related genes (3249 with expression information), we obtained five different subtypes of NSCLC (the inflection points of the lithotripsy plot confirmed 5 as the optimal number of classifications) (**Figures 1A–C**). These NSCLC clusters exhibited significant differences in overall survival (OS, p=0.011), disease-free interval (DFI, p=0.00049), and progression-free interval (PFI, p<0.0001) (**Figure 1D**). Overall, cluster 1 and 5 showed favorable survival, while cluster 2 showed the worst survival (**Figures 1E, F**). Additionally, we assessed the clinical characteristics of patients in different clusters. We found that 69% of patients in cluster 4 were older than 65 years. The majority of patients in clusters 1, 2 and 3 suffered from LUAD, while LUSC predominated in patients in clusters 4 and 5. Besides, a higher proportion of patients in cluster 2 were diagnosed with N2 or N3, T3 or T4, Stage III or Stage IV, indicating that this cluster was characterized by advanced pathological stages, which is consistent with a faster decline in its survival curve (**Figure 1G**). We further investigated enrichment scores of cell death-related pathways between these clusters. We found that cluster 4 showed significantly higher aging-related score, autophagy score, necroptosis score; cluster 5 showed higher extrusion score; cluster 4 and cluster 5 showed significantly higher ferroptosis scores; cluster 2 showed significantly higher NETosis scores; cluster 2 and cluster 4 showed higher pyroptosis scores (**Figures 1H, I**).

### Tumor microenvironment and chemotherapy response in different clusters

**Figure 2:**
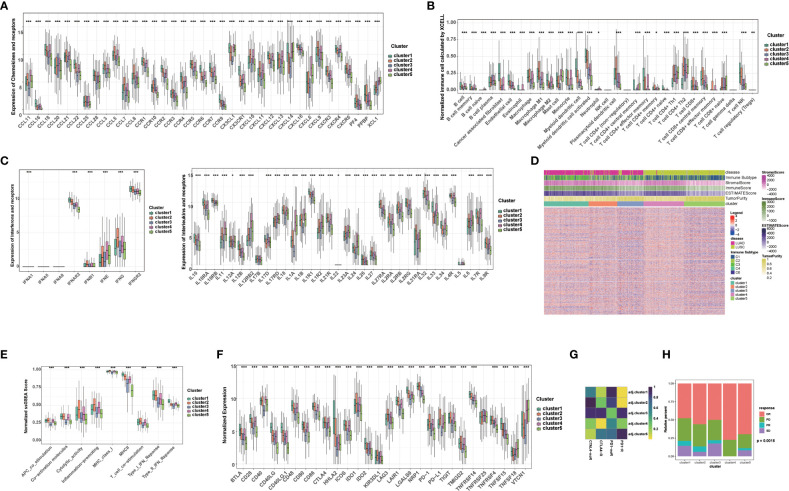
Tumor immune microenvironment and chemotherapy response in different clusters. **(A)** The level of chemokines and their receptors in 5 clusters of NSCLC patients. **(B)** The proportions of immune cells and stromal cells in 5 clusters of NSCLC patients analyzed by xCELL algorithm. **(C)** The level of interferons, interleukins and their receptors in 5 clusters of NSCLC patients. **(D)** The ESTIMATE score, stromal score, immune score, tumor purity, and immune subtype in 5 clusters of NSCLC patients. **(E)** The scores of immune-related functions calculated by GSEA in 5 clusters of NSCLC patients. **(F)** The mRNA expression of immune checkpoints in 5 clusters of NSCLC patients. **(G)** Predicted response rate of different clusters to immune checkpoint inhibitors (CTLA4 and PD1, R: Response, noR: no Response). **(H)** The response to chemotherapy in 5 clusters of NSCLC patients (CR, complete respons; PD, progressive disease; PR, partly response; SD, stable disease). (***p<0.001; **p<0.01; *p<0.05).

Given that immunotherapy is the most promising therapeutic strategy for NSCLC, and cell death plays a significant role in the activation of antitumor immune responses, we analyzed the tumor microenvironment (TME) among the 5 clusters. Tertiary lymphoid structures (TLS) are considered as germinal centers for immune cells in TME, and we assessed the expression of a series of chemokines involved in TLS formation. We found that most chemokines were highly expressed in cluster 1, 2, or 4. Specifically, CCL18, CCL19, CCL22, etc. were highly expressed in cluster 1; CCL3, CCL4, CCL5, etc. were highest in cluster 2; and CCL11, CCL21, CXCL13, etc. was highest in cluster 4 (**Figure 2A**). Overall, the expression of these TIL-involved chemokines was higher in cluster 1, 2, and 4 than in cluster 3 and 5, which regulate immune infiltration in the TME. Consistently, we found cancer-associated fibroblasts (CAFs), endothelial cells (ECs), and most immune cells, such as CD8+ T cells, CD4+ T cells, and antigen-presenting cells, accounted for different proportions of these 5 clusters. In general, they all accounted for a larger proportion in cluster 1, 2, and 4 (**Figure 2B**). In addition, several interferons and their receptors (e.g., IFNE, IFNG, IFNAR2, IFNGR2) and most interleukins and their receptors were associated with immune-activating transcripts. We found that those interferons, interleukins, and their receptors were higher in cluster 1, 2, and 4 than in cluster 3 and 5, which is consistent with higher expression of chemokines and higher infiltration of immune cells in the TME of these clusters (**Figure 2C**).

Subsequently, we further calculated the immune score, stromal score, ESTIMATE score using ESTIMATE algorithm in different clusters. Higher stromal and immune scores indicate higher infiltration of stromal and immune cells, respectively. The ESTIMATE score is the sum of the stromal score and the immune score. Higher ESTIMATE score indicated lower tumor purify. We found that the stromal score, immune score and ESTIMATE score in cluster 1, 2, and 4 were significantly higher than those in cluster 3 and 5, while the tumor purity was lower, indicating that stromal cells and immune cells infiltrated in cluster 1, 2, and 4 (**Figure 2D**). We also analyzed immune-related function scores in each sample, and we found that the most immune-related function scores were lower in cluster 3 and 5, suggesting that these functions were suppressed (**Figure 2E**).

Given that the expression of immune checkpoints is an important basis for immune checkpoints inhibitors (ICIs) therapy, we further evaluated the expression of immune checkpoints in 5 clusters. Notably, the expression of most immune checkpoints (e.g., CD274/PD-L1, CTLA4, PDCD1/PD1, CTLA4, IDO1/2, LAG3) was higher in cluster1, 2, and 4 than in cluster 3 and 5. Among them, PDCD1/PD1 and CD274/PD-L1 were most highly expressed in cluster 2, and CTLA4 was most highly expressed in cluster 2 and 4, suggesting that these clusters may benefit from immunotherapy benefit (**Figure 2F**). Subsequently, we further assessed the response ICIs of different cluster. Cluster 1, 2, and 4 showed better response to anti-PD-1 or CTLA4 antibodies, while cluster 3 and 5 showed relative resistance. Cluster 2 showed the best response to anti-PD-1 antibody, while cluster 4 showed the best response to anti-CTLA4 antibody, which was also consistent with the expression of immune checkpoints (**Figure 2G**). Considering that chemotherapy is the cornerstone of NSCLC treatment, and its use alone or in combination with ICIs is critical for the treatment of NSCLC, we assessed the response to chemotherapy in each cluster. Interestingly, each cluster showed a disease control rate (CR: complete response, PR: partly response, SD: stable disease) of more than 50%, with the highest proportion of CR rate in the cluster 4, suggesting that chemotherapy or chemotherapy combined with ICIs are potential treatments for this cluster (**Figure 2H**). Additionally, cluster 5 was relatively resistant to immunotherapy, but was sensitive to chemotherapy (higher CR+PR ratio) (**Figure 2H**). Taken together, our results suggested that the above-mentioned 5 clusters have relatively distinct TME characteristics. In general, TME in cluster 1, 2, and 4 supported anti-tumor immunity and higher expression of immune checkpoints, which can better benefit from ICIs.

### Enriched pathways, somatic mutations, and DNA methylation patterns of different clusters

**Figure 3:**
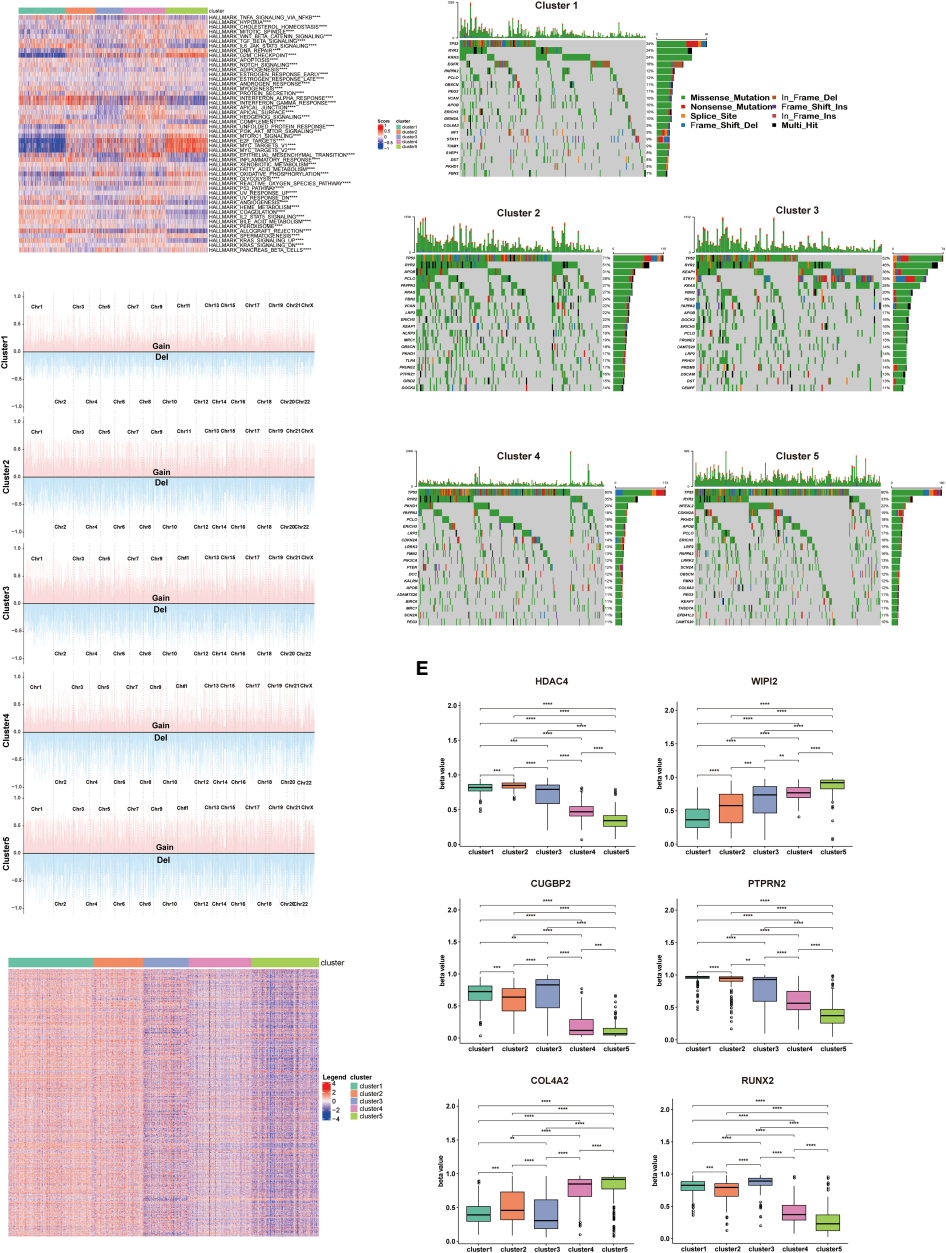
Enriched pathways, somatic mutations, and DNA methylation patterns of different clusters. **(A)** Heatmap showing the differences in hallmarks pathway among 5 NSCLC clusters. **(B)** Waterfall chart showing the somatic mutation frequency and specific mutations of RCD-related genes in 5 NSCLC clusters. **(C)** Display of copy number variation of RCD-related genes in 5 NSCLC clusters. **(D)** Heatmap of differential methylation site of RCD-related genes in 5 NSCLC clusters. **(E)** The methylation level of top differentially methylated genes (HDAC4, WIPI2, CUGBP2, PTPRN2, COL4A2, RUNX2) in 5 NSCLC clusters. (****p<0.0001; ***p<0.001; **p<0.01).

To understand the potential reasons for the distinct prognosis and immune landscapes between these clusters, we investigated related pathway enrichment by GSEA analysis using the hallmark gene set. We found enriched pathways were significantly different in all 5 clusters. Particularly, cluster 1 and 5 showed lower enrichment of oncogenic signaling or targets, such as KRAS signaling, PI3k/Akt/mTOR signaling, E2F target and MYC target, which is consistent with its better prognosis. Furthermore, pro-anti-tumor immunity related pathways, including IL6/JAK/STAT3 signaling, tumor necrosis factor (TNFA, TGFB), interferon (IFNA, IFNG) pathways were enriched in cluster1, 2, and 4, providing a potential explanation for cluster 1, 2, and 4 having a TME supporting anti-tumor immunity and better response to ICIs (**Figure 3A**).

Considering somatic mutations and copy number variation (CNV) as contributors to antitumor immunity and tumor progression (11, 12), we assessed the somatic mutations of cell death related genes in 5 clusters. *TP53* has the highest mutation frequency among these 5 clusters, and cluster 1 showed significantly lower *TP53* mutation frequency compared to other clusters. As the most known tumor suppressor, the mutation of the *TP53* leads to the proliferation and invasion of tumor cells, which is consistent with better survival of cluster 1 patients. Additionally, cluster 2 showed higher *RYR2* mutation frequency compared to other clusters, which has been confirmed to be associated with better survival in NSCLC patients (**Figure 3B**). Detail mutation frequency of each gene was provided in **Table S4**. Likewise, we analyzed the CNV of cell death-related genes in 5 clusters. There were significant differences in CNV of all cell death-associated genes in different clusters, especially in PRKCI, SKIL, SLC2A2, PIK3CA, LAMP3, or TERC. There genes participated in regulating the immune escape and proliferation of NSCLC (13, 14). Additionally, the general CNV frequency of cell death-associated genes in the cluster 1 were slightly lower than that of other clusters (**Figure 3C**). Detail CNV of each gene was provided in **Table S5**.

Since methylation is tightly linked to TME and tumor development (15), we subsequently explored the differential methylation of cell death-associated genes between 5 clusters. 1478 differentially methylated genes and 5260 differential methylation sites were uncovered (**Table S6**). Of note, the methylation degree of *WIPI2* and *COL4A2* in cluster 4 and cluster 5 was significantly higher than that of cluster 1, cluster 2 and cluster 3. In addition, the methylation degree of *HDAC4, CUGBP2, PTPRN2* and *RUNX2* in cluster 1, cluster 2 and cluster 3 was significantly higher than that of cluster 4 and cluster 5, which are involved in regulating the proliferation and invasion of NSCLC (16, 17) (**Figures 3D, E**)

### Establishment of a risk scoring model for NSCLC patients based on cell death-related genes

**Figure 4:**
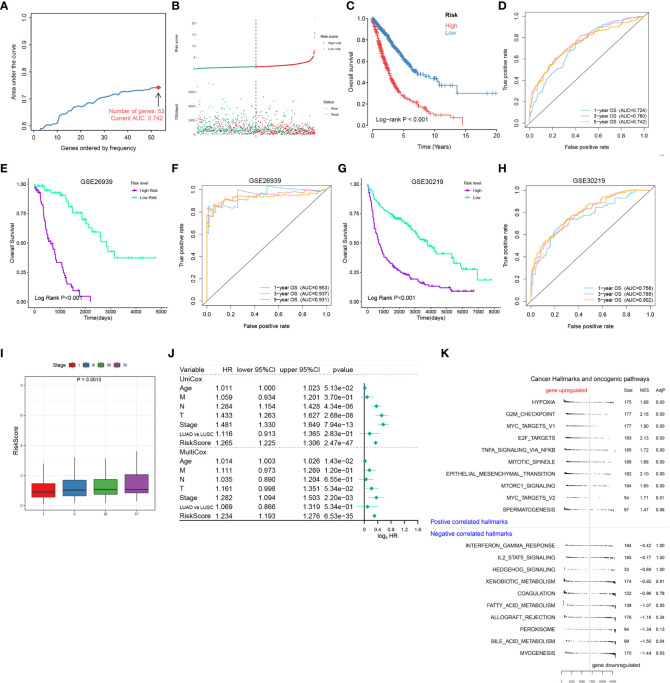
Establishment of a risk scoring model for NSCLC patients based on cell death-related genes. **(A)** Lasso/Cox analysis establishing an RCD-Risk model including 53 RCD-associated genes and the corresponding AUC value. **(B–D)**. Distribution of risk scores and survival status **(B)**, the Kaplan-Meier curve of overall survival **(C)**, or evaluation of prognostic model effectiveness **(D)** between high and low RCD-Risk groups in TCGA database. **(E, F)** the Kaplan-Meier curve of overall survival **(E)**, or evaluation of prognostic model effectiveness **(F)** between high and low RCD-Risk groups in GSE26939. **(G, H)** the Kaplan-Meier curve of overall survival **(G)**, or evaluation of prognostic model effectiveness **(H)** between high and low RCD-Risk groups in GSE30219. **(I)** RCD-risk scores in patients with stage I, II, III, or IV NSCLC. **(J)** Univariate and multivariate Cox analyses evaluate the independent prognostic value of RCD-risk score in TCGA-NSCLC patients. **(K)** The upregulated and downregulated cancer hallmarks and oncogenic pathways in high RCD-Risk group in hallmark gene sets analyzed by GSVA.

The above results confirmed RCD is associated with the prognosis of NSCLC patients and the response of immunotherapy and chemotherapy, suggesting that RCD-related genes may be applied to evaluate prognosis and treatment response. Therefore, we further screened 53 genes among the above RCD-related genes to construct a risk assessment model through LASSO/Cox regression model (**Table S7** and **Figure 4A**). We calculated the risk score for each patient and divided them into high-risk and low-risk groups by the median. The low-risk cohort had a higher number of survivors than the higher-risk cohort (**Figure 4B**). Likewise, Kaplan-Meier analysis also revealed that high-risk patients had poorer OS compared with low-risk patients (**Figure 4C**). Furthemore, the ROC curve showed that this risk model showed satisfactory sensitivity and specificity in predicting survival risk [AUC = 0.724 (1 year), 0.76 (2 years), 0.742 (3 years) (**Figure 4D**)]. The risk model was further validated in GEO datasets GSE26939 and GSE30219. As expected, high-risk patients also showed poor prognosis in the validation dataset. Meanwhile, our model showed satisfactory results in the validation dataset AUC (**Figures 4E–H**), highlighting the stability of this risk model.

Additionally, we also found that risk scores were associated with clinical characteristics of NCSLC patients, such as higher risk score was associated with advanced stages (**Figure 4I**). We further performed a Cox regression model to assess the performance of the risk scoring model in combination with other clinical features (age, pathological type, and pathological stage). We found that in the multivariate Cox model, our risk score model showed better performance compared to age, T stage, N stage, M stage and pathological type (LUAD vs LUSC) (**Figure 4J**). Finally, we performed a GSEA analysis based on differentially expressed genes between high and low risk groups. Pathways associated with treatment resistance and tumor invasion (e.g., Hypoxia, G2M, MYC, EMT) were significantly upregulated in the high-risk group (**Figure 4K**). Taken together, our results suggested that this RCD risk model is a promising and robust biomarker for assessing clinical outcomes in NSCLC patients. Enrichment of pathways associated with treatment resistance and tumor invasion in the high-risk group is potential cause of its poor prognosis.

### RCD-Risk model predicts response to immunotherapy and chemotherapy

**Figure 5:**
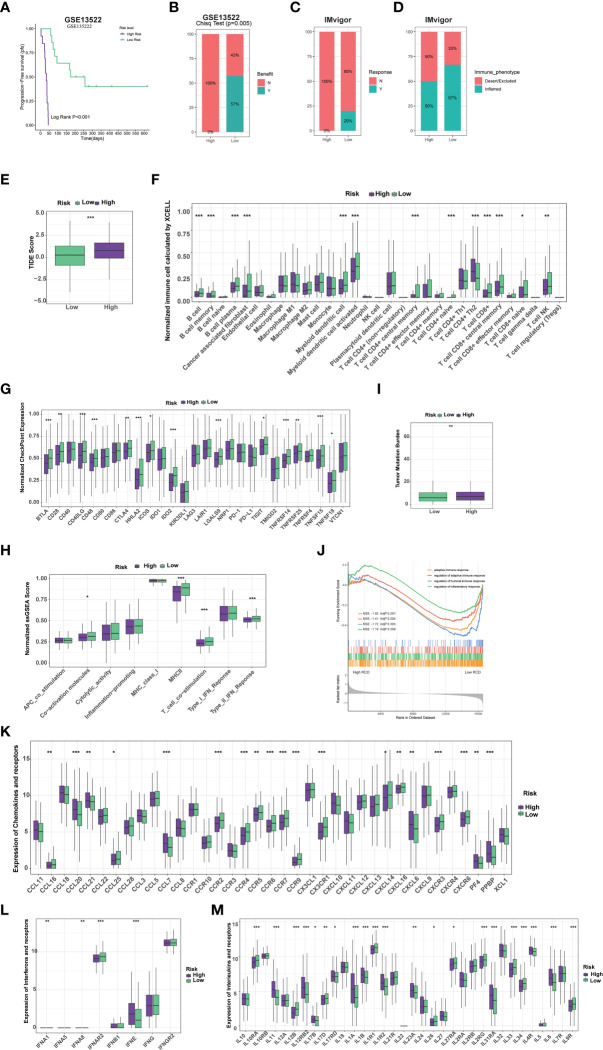
RCD-Risk model predicts response to immunotherapy and chemotherapy. **(A)** The Kaplan-Meier curve for progression-free survival between high and low RCD-Risk NSCLC patients receiving anti-PD-1/PD-L1 therapy in GEO database (GSE135222). **(B, C)** The anti-PD-1/PD-L1 responsiveness between high and low RCD-Risk groups in GSE135222 **(B)** or IMvigor **(C)** dataset. **(D)** The immune phenotypes in high and low RCD-Risk groups in IMvigor dataset. **(E)** Comparison of the tumor immune dysfunction and exclusion (TIDE) prediction scores in the low and high RCD-Risk groups in TCGA database. **(F, G)** The proportions of immune cells and stromal cells **(F)** or the expression of immune checkpoints **(G)** in high and low RCD-Risk groups in TCGA database. **(I, H)** Tumor mutation burden **(I)** or immune-related function scores **(H)** in high and low RCD-Risk groups in TCGA database. **(J)** Gene Ontology (GO) enrichment analysis showing the enrichment of immune responses or inflammatory response in the NCSLC patients with low RCD-Risk in TCGA database. **(K–M)** The level of chemokines **(K)**, interferons **(L)**, interleukins **(M)**, and their receptors in the low and high RCD-Risk groups in TCGA database. (***p<0.001; **p<0.01; *p<0.05).

We further evaluated whether the RCD-Risk model could serve as a predictor of immunotherapy in NSCLC patients. Notably, we found that NSCLC patients in the low-risk group had significantly better prognosis than those in the high-risk group after receiving anti-PD-1/PD-L1 therapy (GSE135222, **Figure 5A**). None of the patients in the high-risk group responded to anti-PD-1/PD-L1 therapy, while more than half of the patients in the low-risk group responded to anti-PD-1/PD-L1 therapy (**Figure 5B**). We analyzed NSCLC patient data in the IMvigor210 cohort, and we found that patients in the low-risk group responded better to anti-PD-L1 therapy than those in the high-risk group (**Figure 5C**). Interestingly, the immune manifestations of patients in the high-risk group were dominated by Desert/Excluded, while those in the low-risk group were dominated by Inflamed, which explained why patients in the low-risk group could benefit from immunotherapy (**Figure 5D**). We than further used tumor immune dysfunction and exclusion (TIDE) score to verify the superiority of the RCD-Risk model in predicting the efficacy of immunotherapy. We found that TIDE scores were significantly higher in high-risk patients than in low-risk patients, also suggesting that low-risk patients could better benefit from immunotherapy (**Figure 5E**).

To understand the differences in response to immunotherapy between high and low risk groups, we assessed immune cell infiltration, immune checkpoint expression, and immune function in each group (**Figures 5F–H**). We found that stromal cells, such as cancer-associated fibroblasts (CAFs), most immune cells, such as CD8+ T cells, CD4+ T cells, and antigen-presenting cells, were significantly higher in the low-risk group than in the high-risk group (**Figure 5F**). Similarly, the expression of most immune checkpoints and many immune-related function scores were higher in the low-risk group, which partly explains that patients in the low-risk group benefited more from immunotherapy (**Figures 5G, H**). Previous studies have shown that tumor mutational burden (TMB) is inversely related to the response of immunotherapy (18). We found that TMB in patients in the low-risk group was lower than that in the high-risk group, suggesting that the RCD-Risk model may evaluate the prognosis of NSCLC patients with immunotherapy independently of TMB, highlight the effectiveness of the model (**Figure 5I**). We further performed GSEA analysis. The GO terms “adaptive immune response”, “regulation of adaptive immune response”, “regulation of humoral immune response”, and “regulation of inflammatory response” were enriched in low RCD-risk group (**Figure 5J**). In addition, the levels of many important chemokines, interferons, interleukins, and their receptors were higher in low RCD-risk group than in high RCD-risk group (**Figure 5K–M**), which interprets the dramatical benefit of immunotherapy for NSCLC patients with low RCD-risk scores.

In view of the important role of chemotherapy combined with immunotherapy in the treatment of NSCLC patients, we also evaluated the difference in response to chemotherapy in high- and low-risk groups. We found that patients in the low-risk group were more sensitive to Gefitinib, Staurosporine, Erlotinib, etc. (**Figure S1A**), while those in the high-risk group were more sensitive to Sorafenib, Crizotinib, Paclitaxel (**Figure S1B**). We further used Connectivity Map (CMap) to predict drug treatment. Drugs with lower scores mean higher reversal potency and greater potential for application. We predicted that exisulind, a selective apoptotic antineoplastic drug, might have greater therapeutic value for patients in the high-risk group (**Figure S1C**). In conclusion, the RCD-based risk model is not only a reliable indicator for predicting the prognosis of NSCLC patients with immunotherapy, but also an effective biomarker for predicting the sensitivity of patients to chemotherapeutic drugs.

### LDLRAD3 expression is associated with the prognosis of patients with NSCLC

To further verify the performance of RCD-Risk model in a clinically translatable tool, we further investigated the RCD-associated genes applied in RCD-Risk model. Most of them has been confirmed to be associated with survival of different type of cancers; however, LDLRAD3 was rarely reported associated with cancer progression. LDLRAD3 encodes Low Density Lipoprotein Receptor Class A Domain Containing 3 and acts upstream of or within receptor-mediated endocytosis and regulation of protein processing (19). Previous studies have reported circ-LDLRAD3, which is derived from the exon5 region of LDLRAD3 mRNA by “back-splicing,” was associated with the progression of pancreatic cancer (20). However, rare research reported the role of LDLRAD3 mRNA in cancers.

**Figure 6:**
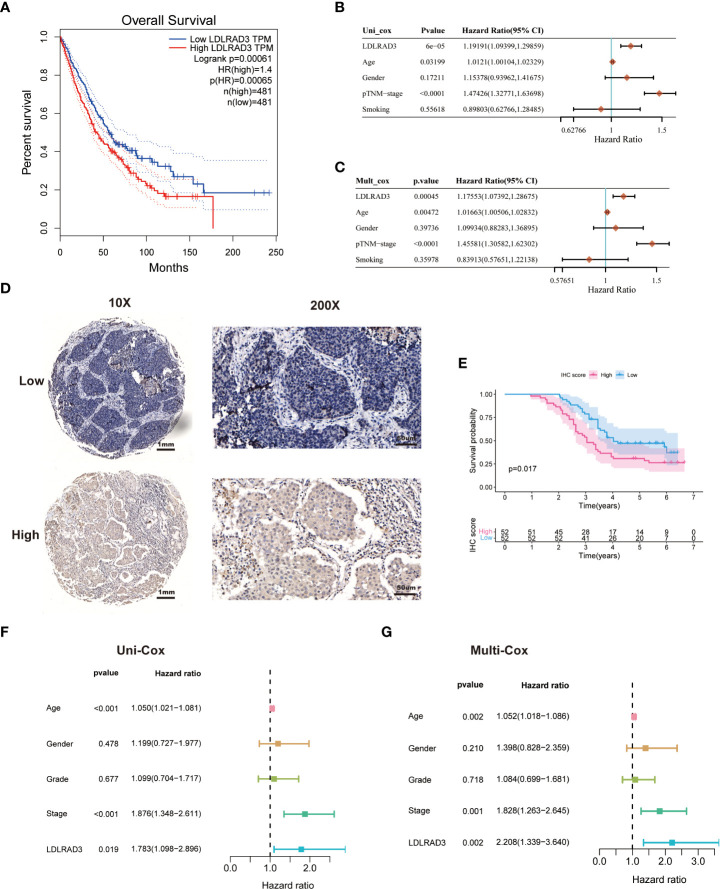
LDLRAD3 expression is associated with the prognosis of patients with NSCLC. **(A)** The Kaplan-Meier curve for overall survival in TCGA-NSCLC patients with high or low LDLRAD3 expression. **(B, C)** Univariate and multivariate Cox analyses evaluate the independent prognostic value of LDLRAD3 expression in TCGA-NSCLC patients. **(D)** Representative IHC staining of LDLRAD3 among 104 NSCLC samples. **(E)** The Kaplan-Meier curve for overall survival in 104 NSCLC samples with high or low LDLRAD3 expression. **(F, G)** Univariate and multivariate Cox analyses evaluate the independent prognostic value of LDLRAD3 expression in 104 NSCLC samples.

We found that high expression of LDLRAD3 was associated with deteriorating survival of NSCLC patients in the TCGA database performed by Kaplan-Meier analysis (**Figure 6A**). Similarly, univariate Cox regression model revealed that LDLRAD3 expression significantly correlated poor prognosis (**Figure 6B**); after controlling for confounding variables (including age, gender, TNM stage, and smoking), LDLRAD3 expression remained statistically significant for OS (**Figure 6C**). Notably, we confirmed that high expression of LDLRAD3 was correlated with inferior survival using immunohistochemical staining in NSCLC samples (**Figures 6D, E**); and consistently, uni- and multi-Cox regression demonstrated LDLRAD3 expression as an independent prognostic factor (**Figures 6F, G**), further supporting the robustness and stability of our RCD-Risk model in NSCLC.

### Overexpression of LDLRAD3 promotes the proliferation and invasion abilities of NSCLC

**Figure 7:**
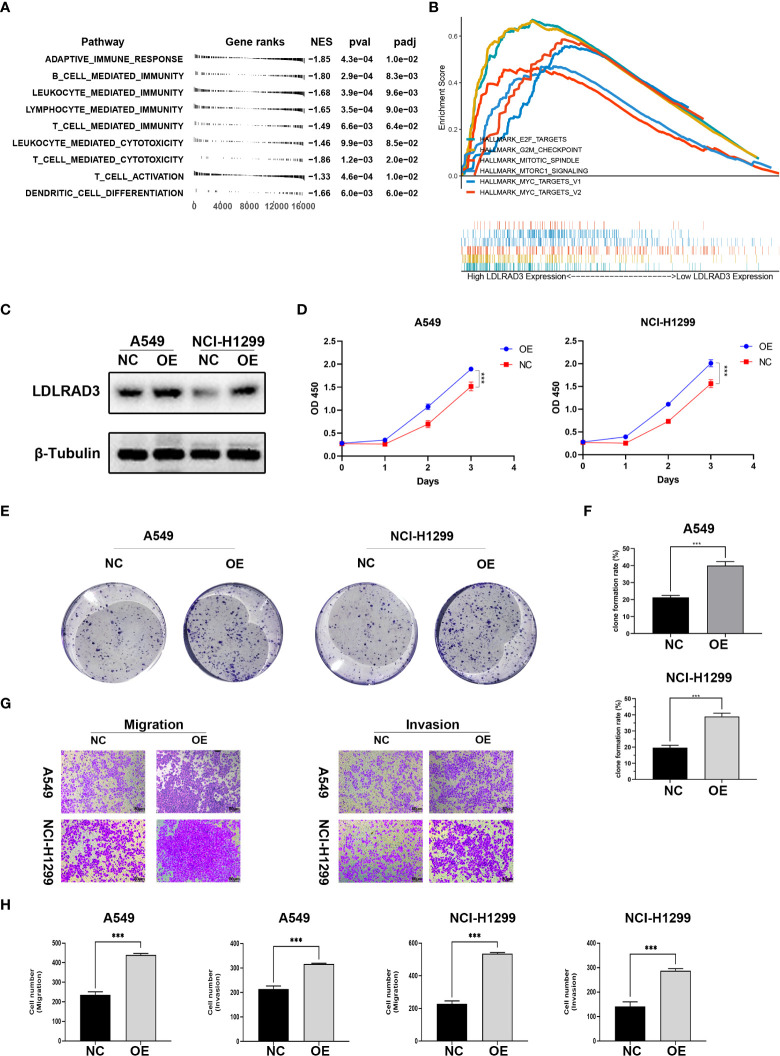
Overexpression of LDLRAD3 promotes the proliferation and invasion abilities of NSCLC. **(A)** Gene Ontology (GO) analysis showing the normalized enrichment scores (NES) of adaptive immune response and immune cell-related pathways in the NCSLC patients with high LDLRAD3 expression. The negative NES indicating a negative correlation between the corresponding pathway and LDLRAD3 expression. **(B)** GSEA analysis showing the enrichment of oncogenic signaling or process (including MYC targets, MTORC1 signaling, E2F targets, G2M checkpoints, and mitotic spindle) in NCSLC patients with high LDLRAD3 expression. **(C)** Overexpression (OE) of LDLRAD3 in A549 and H1299 validated by Western Blotting analysis. **(D)** The proliferation of LDLRAD3 overexpressed cells (OE) and control cells (NC) determined by CCK-8 assay. **(E, F)**. Clone formation ability of indicated NSCLC cells. **(G, H)**. Migration and invasion abilities of indicated NSCLC cells determined by transwell assay. (***p<0.001).

To further determine the role of LDLRAD3 in TME and progression of NSCLC, we first analyzed the involvement of LDLRAD3 in TME of NSCLC patients (TCGA) using GO database. The expression of LDLRAD3 was negatively correlated with adaptive immune response, DC cell differentiation, T cell activation, and immune cell-mediated immunity and cytotoxicity (**Figure 7A**). Consistently, the expression of LDLRAD3 was negatively correlated with MHC complex, immune receptor, and immunoglobulin (**Figure S2A**). We further compared the levels of multiple cytokines, HLA molecules, and immune checkpoints between LDLRAD3-high and -low groups. Similarly, LDLRAD3-low group mostly increased cytokines, HLA molecules and immune checkpoints compared to LDLRAD3-high group, suggesting that LDLRAD3 play an immunosuppressive role in TME of NSCLC (**Figure S2B**).

For the involvement of LDLRAD3 in progression of NSCLC, the GSEA analysis showed that many oncogenic signaling or process were enriched in patients with high LDLRAD3 expression, such as MYC targets, MTORC1 signaling, E2F targets, G2M checkpoints, and mitotic spindle, suggesting that LDLRAD3 is involved in the progression of NSCLC (**Figure 7B**). Subsequently, we overexpressed LDLRAD3 in A549 and H1299 cells, respectively (**Figure 7C**). As expected, overexpression of LDLRAD3 significantly promoted the proliferation (**Figure 7D**), clone formation (**Figures 7E, F**), migration and invasion abilities (**Figures 7G, H**), consistent with the GSEA analysis.

## Discussion

Immunotherapy represented by ICIs has become a second-line standard treatment scheme for advanced NSCLC, and its indications for first-line treatment are gradually being approved. Immunotherapy for lung cancer is moving towards early neoadjuvant treatment. After breakthrough progress in immunotherapy, immunotherapy combined with radiotherapy, chemotherapy and dual immunotherapy have further improved the efficacy of patients. Overall, immunotherapy has significant effect in the field of NSCLC, and has expanded to the field of small cell lung cancer, benefiting more and more patients. At the same time, immunotherapy for lung cancer faces many challenges and the main one is how to screen the beneficiaries of lung cancer immunotherapy. In this research, we classified 999 NSCLC in the TCGA database into five clusters based on RCD-associated genes. Significant differences of survival were observed in different clusters. Among them, cluster 5 showed best survival, whereas cluster 2 showed worst survival. Additionally, these clusters also showed different enriched pathways, including cell cycle associated pathways (G2/M checkpoint, Myc targets, eIF2 targets), tumor angiogenesis pathways (angiogenesis, hypoxia), immune environment pathways (TNF-α, interferon response), and so on. All these different pathways interacted with cell death in NSCLC, thus regulating cancer development (21–23). Furthermore, we also demonstrated a series of epigenetic differences between different clusters. Cluster 2/4/5 showed higher *TP53* mutation frequency. Several clinical studies have confirmed that NSCLC patients with *TP53* mutation indicated poor prognosis and was relatively more resistant to chemotherapy and radiation therapy (24, 25). Patterns of DNA methylation are critical for gene regulation, transposon silencing, and gene imprinting. We disclosed significant difference of DNA methylation patterns between different clusters. WIPI2, COL4A2, HDAC4, CUGBP2, PTPRN2 and RUNX2 are Top 6 differential methylation genes, and all of them were reported to take part in regulation of cancers. For instance, WIPI2 regulates the proliferation of hepatocellular cancer cells through the AMPK signaling pathway (26). HDAC4 regulates apoptosis in NSCLC treated with synthetic retinoid (27).

Immune cells in the tumor microenvironment (TME) have been proven to be crucial in the development of various tumors. Different types of tumors have different immune cell subpopulations. Even for the same pathological type, the subpopulation could be different among patients (28, 29). We found cluster 5 showed higher infiltration of Tfh, but lower M2 macrophages. Oppositely, cluster 2 showed lower Tfh, but higher M2 macrophages. Tfh cells supported the maturation of tumor-adjacent tertiary lymphoid structures, thus promoting anti-tumor immunity (30). M2 macrophage promotes gene instability, angiogenesis, fibrosis, immunosuppression, invasion and metastasis to enhance tumor progression (31). Additionally, cluster 2 showed higher infiltration of CAFs and ECs, whereas cluster 5 showed lower infiltration. CAFs secret a variety of growth factors, chemokines and proteases, thus regulating development and invasion of cancer cells (32). Similarly, abundant tumor ECs provide nutrients and energy for the rapid proliferation of cancers. These differences in immune infiltration tally with the difference in survival. Immune checkpoints inhibitors are a promising therapeutic strategy for NSCLC, especially for chemotherapy-resistant patients (33). High expression of immune checkpoints promotes immune escape of cancers, and indicated better response to immune checkpoints inhibitors. We found several main immune checkpoints were all downregulated in cluster 5, but upregulated in cluster 2. Notably, Cluster 1, 2, and 4 showed better response to ICIs, which should be due to TME supporting anti-tumor immunity, including IL6/JAK/STAT3 signaling, tumor necrosis factor (TNFA, TGFB) and interferon (IFNA, IFNG) pathways (21, 34, 35).

Furthermore, we constructed a 53 genes risk scoring model for predicting prognosis of NSCLC. The model showed satisfactory AUC in both training group (TCGA database) and test group (2 GEO datasets). In Kaplan-Miler analysis, high-risk score NSCLC patients showed significantly poor survival compared with low-risk score NSCLC patients. Furthermore, we also confirmed the constructed risk score was an independent predicted factor in both univariate and multivariate Cox regression analysis. Our constructed risk model showed advantages in predicting survival compared with traditional clinical and pathological features. Finally, we also confirmed LDLRAD3, which was rare reported in previous studies of cancers, was associated with worse survival of NSCLC patients. Moreover, overexpression of LDLRAD3 also significantly promoted the proliferation and invasion abilities of NSCLC, which maybe a potential therapeutic target for NSCLC.

In summary, our research revealed the landscape of genomic alterations and immune infiltration based on RCD-associated genes. Remarkably, we developed a stable and potent RCD-risk model for assessing the prognosis and immunotherapy benefit, representing a promising tool to optimize decision-making and surveillance protocols for individual NSCLC patients. One of the main disadvantages for the research is lack of the data from multicenter trials. We will further collect data to validate and develop our RCD-risk model.

## Data availability statement

The raw data supporting the conclusions of this article will be made available by the authors, without undue reservation.

## Author contributions

GL and TH designed this project. ZY and WS performed the bioinformatics analysis and experiments. GL, BF, and TH wrote the manuscript and performed the data review. GL supervised the project, established the collaboration, and provided funding support for the project. All authors read and approved the manuscript. All authors contributed to the article and approved the submitted version.

## Supplementary material

Supplementary Figure 1 RCD-Risk model predicts response to chemotherapy and targeted therapy. **(A)** Favorable drugs and their sensitivity in the low RCD-Risk group in TCGA database. **(B)** Favorable drugs and their sensitivity (or IC_50_) in the high RCD-Risk group in TCGA database. **(C)** Favorable drugs for high RCD-Risk populations were predicted by Connectivity Map (CMap) using eXtreme Sum (XSum) algorithm. The blue dots represent drugs that benefit high-risk populations, where scores are negatively correlated with the significance of the drug in the high-risk population. Exisulind is the most beneficial drug for patients in the high-risk group in this prediction.

Supplementary Figure 2 RCD-Risk model predicts response to chemotherapy and targeted therapy. **(A)** Gene Ontology (GO) analysis showing the normalized enrichment scores (NES) of immune-related pathways in the NCSLC patients with high LDLRAD3 expression. The negative NES indicating a negative correlation between the corresponding pathway and LDLRAD3 expression. **(B)** Heatmap showing the level of chemokines, interferons, interleukins, their receptors, MHC molecules, and immune checkpoints in TCGA-NSCLC patients with high or low LDLRAD3 expression.
