# Supplementary figures and images for: A unique regulated cell death-related classification regarding prognosis and immune landscapes in non-small cell lung cancer
Source: Front Immunol. 2023 Feb 3;14:1075848. doi: 10.3389/fimmu.2023.1075848 (PMC9936314; doi:10.3389/fimmu.2023.1075848)

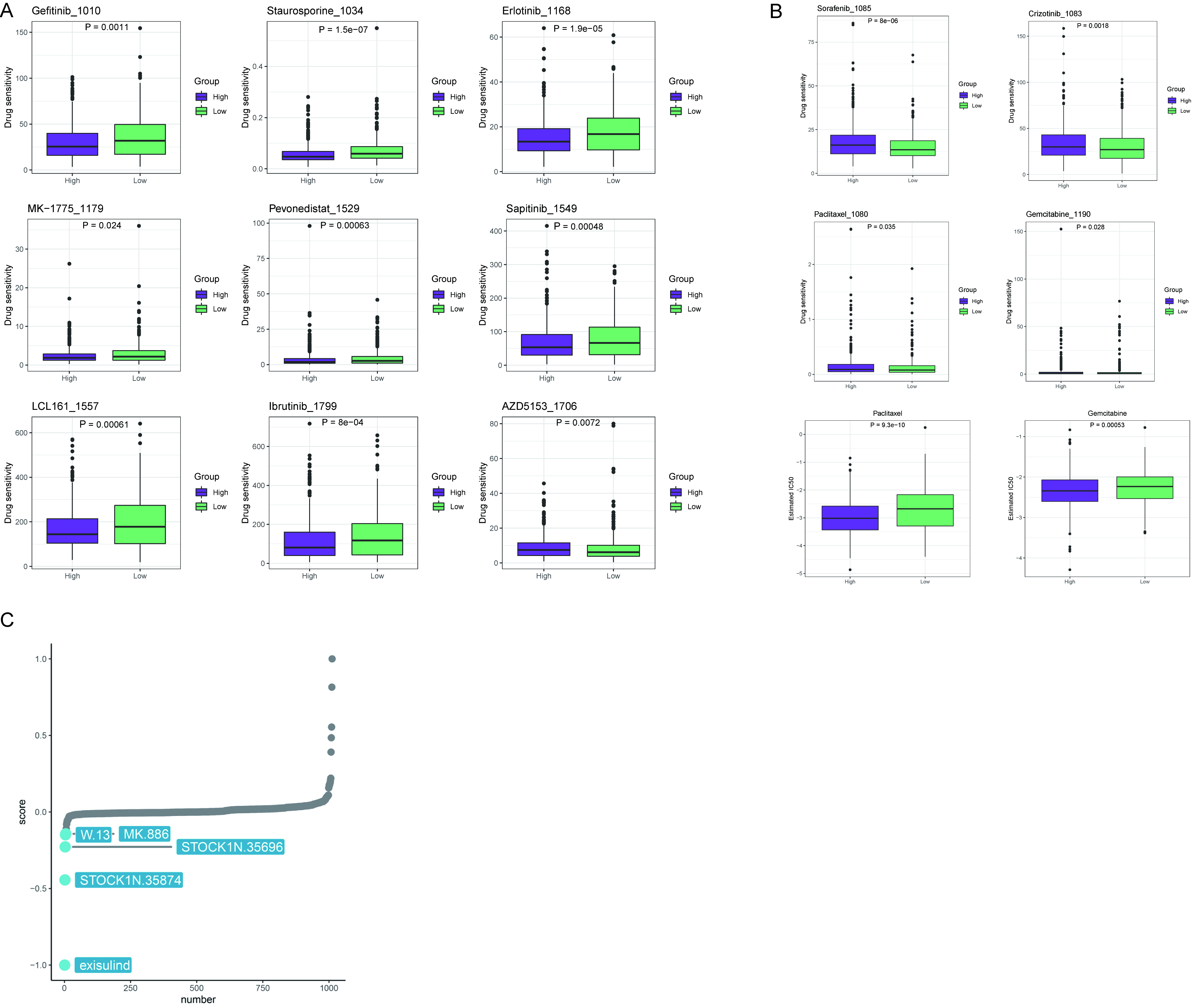

Supplement: Supplementary Figure 1 — RCD-Risk model predicts response to chemotherapy and targeted therapy. (A) Favorable drugs and their sensitivity in the low RCD-Risk group in TCGA database. (B) Favorable drugs and their sensitivity (or IC50) in the high RCD-Risk group in TCGA database. (C) Favorable drugs for high RCD-Risk populations were predicted by Connectivity Map (CMap) using eXtreme Sum (XSum) algorithm. The blue dots represent drugs that benefit high-risk populations, where scores are negatively correlated with the significance of the drug in the high-risk population. Exisulind is the most beneficial drug for patients in the high-risk group in this prediction. [file Image_1.tif]

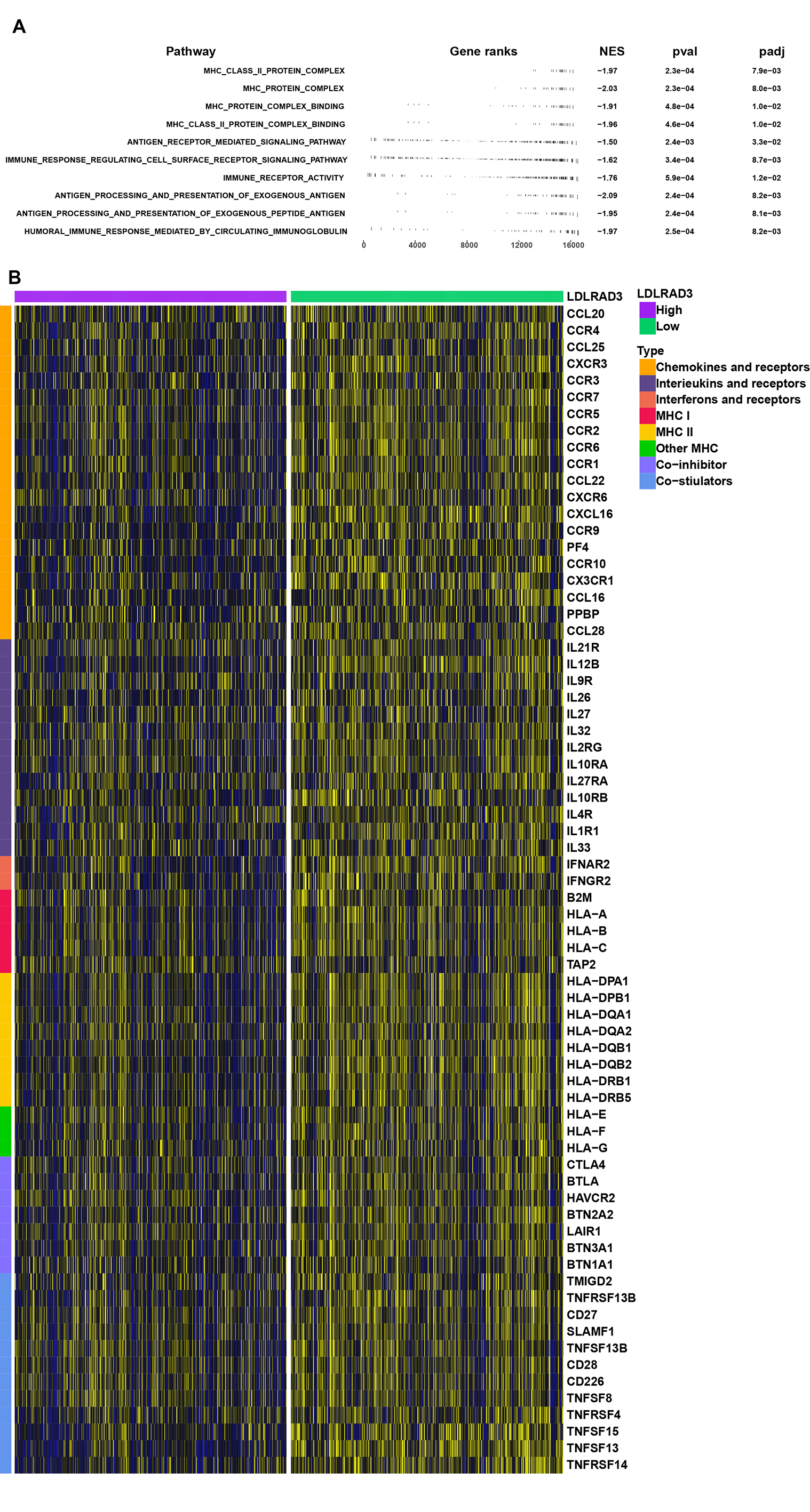

Supplement: Supplementary Figure 2 — RCD-Risk model predicts response to chemotherapy and targeted therapy. (A) Gene Ontology (GO) analysis showing the normalized enrichment scores (NES) of immune-related pathways in the NCSLC patients with high LDLRAD3 expression. The negative NES indicating a negative correlation between the corresponding pathway and LDLRAD3 expression. (B) Heatmap showing the level of chemokines, interferons, interleukins, their receptors, MHC molecules, and immune checkpoints in TCGA-NSCLC patients with high or low LDLRAD3 expression. [file Image_2.tif]
