# Supplementary material for: A unique regulated cell death-related classification regarding prognosis and immune landscapes in non-small cell lung cancer
Source: Front Immunol. 2023 Feb 3;14:1075848. doi: 10.3389/fimmu.2023.1075848 (PMC9936314; doi:10.3389/fimmu.2023.1075848)
Supplement: Supplementary file 3 [file Table_1.docx]

Table S1. Clinical features of 999 cases NSCLC patients in the TCGA database

|  | Tumor |
| --- | --- |
| Age |  |
| >65 | 557 |
| <=65 | 427 |
| NA | 15 |
| stage |  |
| Stage I | 513 |
| Stage II | 278 |
| Stage III | 164 |
| Stage IV | 32 |
| unknown | 12 |
| Pathologic_M |  |
| M0 | 742 |
| M1 | 31 |
| MX | 226 |
| pathologic_N |  |
| N0 | 643 |
| N1 | 222 |
| N2 | 110 |
| N3 | 7 |
| NX | 17 |
| pathologic_T |  |
| T1 | 283 |
| T2 | 556 |
| T3 | 115 |
| T4 | 42 |
| TX | 3 |
| disease |  |
| LUAD | 505 |
| LUSC | 494 |
| cluster |  |
| cluster1 | 247 |
| cluster2 | 158 |
| cluster3 | 145 |
| cluster4 | 224 |
| cluster5 | 225 |
| Immune Subtype |  |
| C1 | 351 |
| C2 | 324 |
| C3 | 184 |
| C4 | 25 |
| C6 | 41 |
| NA | 74 |
| OS |  |
| Alive | 604 |
| Dead | 395 |
| DFI |  |
| Alive | 450 |
| Dead | 150 |
| NA | 399 |
| PFI |  |
| Alive | 646 |
| Dead | 353 |
