# Supplementary material for: A unique regulated cell death-related classification regarding prognosis and immune landscapes in non-small cell lung cancer
Source: Front Immunol. 2023 Feb 3;14:1075848. doi: 10.3389/fimmu.2023.1075848 (PMC9936314; doi:10.3389/fimmu.2023.1075848)
Supplement: Supplementary file 4 [file Table_2.docx]

Table S2. Clinical features of NSCLC patients in the GEO database

|  | GSE26939 | GSE30219 |
| --- | --- | --- |
| event (OS) |  |  |
| Alive | 49 | 58 |
| Dead | 66 | 88 |
| ALL | 115 | 146 |
